# The first patient with a pure 1p36 microtriplication associated with severe clinical phenotypes

**DOI:** 10.1186/s13039-014-0064-9

**Published:** 2014-10-03

**Authors:** Fang Xu, Ya-Nan Zhang, De-Hua Cheng, Ke Tan, Chang-Gao Zhong, Guang-Xiu Lu, Ge Lin, Yue-Qiu Tan

**Affiliations:** Institute of Reproduction and Stem Cell Engineering, Central South University, 110# Xiangya Road, Changsha, Hunan 410078 PR China; Reproductive and Genetic Hospital of Citic-Xiangya, Changsha, Hunan 410078 PR China; National Engineering and Research Center of Human Stem Cell, Changsha, 410078 PR China

**Keywords:** Copy number variations, 1p36 microtriplication, 1p36 microdeletion, Single nucleotide polymorphism microarray

## Abstract

**Background:**

Copy Number Variants (CNVs) is a new molecular frontier in clinical genetics. CNVs in 1p36 are usually pathogenic and have attracted the attention of cytogeneticists worldwide. None of 1p36 triplication has been reported thus far.

**Results:**

We present three patients with CNVs in 1p36. Among them one is the first 1p36 tetrasomy due to a pure microtriplication and the other two are 1p36 microdeletion. Traditional chromosome G-banding technique showed a normal karyotype. Single nucleotide polymorphism (SNP) microarray analysis combined with multiplex ligation-dependent probe amplification (MLPA) and fluorescence in situ hybridization (FISH) were used to identify and confirm the chromosome microdeletion/microtriplication. The facial dysmorphisms of the patient with 1p36 tetrasomy differed from those two patients with 1p36 monosomy. The expression levels of *B3GALT6*, *MIB2*, *PEX10* and *PANK4* in the blood were determined, and differential expressions were observed between the patients and controls.

**Conclusions:**

Our study shows the first case of 1p36 tetrasomy due to a pure microtriplication in a patient with severe intellectual disability and seizures. The study provides a new resource for studying the mechanisms of microtriplication formation, and provides an evidence that overexpression of the specific genes might be related the specific phenotype of 1p36 microtriplication.

**Electronic supplementary material:**

The online version of this article (doi:10.1186/s13039-014-0064-9) contains supplementary material, which is available to authorized users.

## Background

Copy Number Variants (CNVs) is a new molecular frontier in clinical genetics [1]. Some CNVs are associated with genomic disorders with extreme phenotypic heterogeneity, which brings challenges in genetic diagnosis, counseling, and management [2]. CNVs in 1p36 include microdeletion and microduplication. 1p36 microdeletion is relatively common, with an incidence of ~1 in 5000 newborns [3,4]. Its main phenotypes include intellectual disability, developmental delay, prominent forehead, low-set ears, long philtrum, pointed chin, short feet, hearing loss, seizures, hypomyotonia, feeding difficulties, speech delay, strabismus, heart defects, obesity, skeletal anomalies, renal and genital abnormalities [3,4].

In contrast, 1p36 microduplication is rare, with only two cases of “pure” 1p36 microduplication having been reported, one affecting a 6-month-old male infant and the other, a 17-year-old male patient [5]. The major phenotypic features in these two patients were hypotonia, severe psychomotor delay, intellectual disability, developmental delay and speech defects.

Here, we present three patients with CNVs in 1p36. Among them one is the first 1p36 tetrasomy due to a pure microtriplication and the other two are 1p36 microdeletion. Changes in chromosome copy numbers were detected and confirmed by SNP array, MLPA and FISH analyses. Expression levels of 4 genes (*B3GALT6*, *MIB2*, *PEX10* and *PANK4*) mapping to the deleted and triplicated segments were determined by quantitative PCR analysis, which showed that transcript levels correlated with altered DNA copy numbers, indicating that the observed phenotypes resulted from gene-dosage effects.

## Case presentation

**Figure 1:**
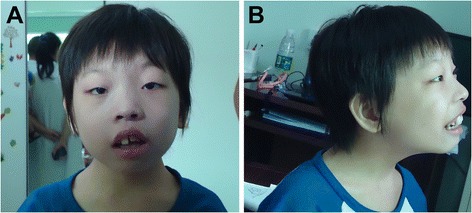
**Clinical photographs of the patient with 1p36 triplication.** **(A)** Front view of the face. **(B)** Lateral view of the face. The major facial phenotype included strabismus, hypertelorism, low hairline, ear malformations, broad nasal bridge, wide mouth, thick lips and prominent incisors.

An eight-year-old female patient was brought to our clinic by her non-sanguineous parents due to severe intellectual disability and seizures. The patient, the first and only child of a 24-year-old father and a 22-year-old mother, was born at full term by a normal delivery after an uneventful pregnancy. She had suffered from feeding problems in infancy as well as a medical history of severe seizures. On top of that, she could also catch a cold and fever very easily, and had once been hospitalized because of pneumonia. She developed delay obviously at each developmental milestone, including gross motor, fine motor, language, cognitive development and social behavior. For instance, she was capable of walking until 2 years and half and saying “dada” and “mama” until 2 years. She undergone surgery for congenital left eyelid ptosis at the age of 3 years. Now she was 8 years old, and according to our physical examination, her weight, height and body mass index (BMI) represented were 18 kg, 108.5 cm, and 15.29 kg/m^2^ (25th–50th centiles) [6], respectively; Also, she has got microcephaly (head circumference, 49.6 cm; reference value, 52.19 cm). Facial dysmorphism, with strabismus, hypertelorism, low hairline, ear malformations, broad nasal bridge, wide mouth, thick lips and prominent incisors could be observed (Figure 1; Table 1). The child could understand some simple sentences, whose expressive ability to speak simple words and sentences is extremely poor. It can be seen that she had behavioral abnormalities, unable to take care of herself, no to mention getting focused and controlling herself well. However, ultrasonography of the uterus, liver, gallbladder, spleen, pancreas, kidneys and heart showed no abnormalities.

**Table 1:** **Features presented in patients with 1p36 micro-rearrangement**

	**1p36 deletion**			**1p36 duplication (Giannikou et al., 2012) [** 5 **]**	1p36 triplication*
	**Bursztejn et al., 2009 [** 7 **]**	Case 1*	Case 2*		
Mental retardation	52/60	+	+	+	+
Developmental delay	60/60	+	+	+	+
Speech defects	60/60	+	+	+	+
Behavioral problems	28/60	+	+	+	+
Hypotonia	?	+	+	+	+
Feeding difficulty	?	+	+	+	+
Microbrachycephaly	52/60	+	+	?	+
Low hairline	?	+	+	?	+
Prominent forehead	?	+	+	?	+
Strabismus	?	+	_	?	+
Hypertelorism	?	+	+	?	+
Low-set ears	?	+	+	+	+
Ear malformations	?	_	+	+	+
Broad nasal bridge	60/60	+	+	+	+
Long philtrum	60/60	_	_	+	+
Thick lips	?	_	_	_	+
Pointed chin	60/60	+	+	+	_
Wide mouth	?	_	_	_	+
Prominent incisors	?	_	_	+	+
Short feet	48/60	+	_	+	+
Hearing loss	52/60	_	_	_	_
Seizure	26/60	+	_	+	+
Obesity	?	+	+	+	_
Skeletal anomalies	13/32	_	+	_	_

*: presented in this study.

“+” : present; “_”: absent; “?”: uncertain.

Other two 8-year-old female patients with 1p36 microdeletion syndrome were also identified in the study by FISH and MLPA techniques (Additional file 1).

### Results

**Figure 2:**
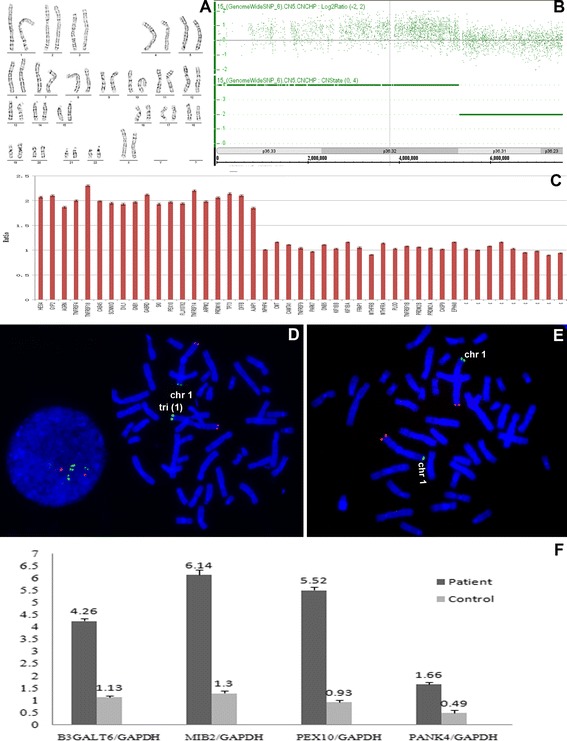
**Copy number variants analysis of the patient: (A) “Normal” high-resolution karyotype.** **(B)** A SNP microarray analysis detected an approximately 5.28-Mb triplication in the short arm of chromosome 1. **(C)** MLPA result using SALSA kit P147-B1 of MRC Holland, showing a triplication included all probes from *ISG15* up to *AJAP1*(RPH are approximately 2.0). **(D)** FISH results of the patient. Two red signals with the similar strength were presented in the long arm of chromosome 1 by the control probe TelVysion 1q. However, by the testing probe TelVysion 1p, the green hybridization signal in one 1p was obviously stronger than that in the other 1p, and in interphase nucleus, four green signals could be seen. **(E)** FISH image of the parents of the patient showed normal appearance, indicating that the 1p partial triplication is *de novo* (the FISH image of mother didn’t shown). **(F)** Quantitative PCR analysis showed the patient had 3 to 6 times as higher expression levels than control for the four genes *B3GALT6*, *MIB2*, *PEX10*, *PANK4* in the blood.

Karyotype analysis at 550 band resolution showed the proband with a female karyotype(46,XX), without any suggestion of chromosome abnormality (Figure 2A). Her parents also presented normal karyotype.

Single nucleotide polymorphism (SNP) microarray analysis by Affymetrix SNP 6.0 was performed as our previous report [8,9]. The result showed a pure 5.28 Mb microtriplication at 1p36.33p36.32, and the karyotype could be expressed as 46,XX.arr[hg19]1p36.33p36.32(1–5,280.000) × 4 (Figure 2B), which was considered as a pathogenic CNV according to the available databases including Database of Genomic Variant (http://projects.tcag.ca/variation/), DECIPHER (https://decipher.sanger.ac.uk/), ECARUCA (http://umcecaruca01.extern.umcn.nl:8080/ecaruca/ecaruca.jsp), OMIM (http://www.omim.org/), and pubmed (http://www.pubmed.gov/).

Multiplex ligation dependent probe amplification (MLPA) analysis using the SALSA kit P147-B1 (MRC-Holland, Amsterdam, the Netherlands, see http://www.mlpa.com) confirmed the microtriplication for chromosome 1p (Figure 2C), including all probes from *ISG15* up to *AJAP1*, which was consistent with the SNP microarray result. The SNP array result was also confirmed by Fluorescent in situ hybridization (FISH) with two subterminal probes TelVysion 1p (SpectrumGreen labeled) and TelVysion 1q (SpectrumOrange labeled) which were purchased from Abbott-Vysis, Inc (Downers Grove, IL, USA) (Figure 2D and 2E).

Quantitative PCR analysis showed that transcript levels in the blood correlate with altered DNA copy numbers. For the selected 4 OMIM genes *B3GALT6* (betaGal beta 1,3-galactosyltransferase polypeptide 6), *MIB2* (mindbomb E3 ubiquitin protein ligase 2), *PEX10* (peroxisomal biogenesis factor 10), *PANK4* (pantothenate kinase 4), which are situated in the 1p36.33p36.32 region and were expression in blood, the ratios of the patient to the controls (a healthy 8-year-old female sample) showed significant differences (Figure 2F), indicating upregulated and deregulated expression respectively.

### Discussion

Here, we report the first case of a pure 1p36.33p36.32 tetrasomy resulting from a de novo microtriplication detected on SNP microarray and confirmed by MLPA and FISH analyses. Our results provide a new case for exploring the formation mechanism of 1p36 microtriplication and the molecular etiology of the patient.

The most common mechanism underlying CNVs is non-allelic homologous recombination and not non-homologous end joining mediated by low copy repeats during meiosis [10]. Cytogenetically invisible tetrasomy due to a chromosome segment microtriplication is a very complex and rare CNV. Till date, only a few triplications have been reported, including 2q11.2 → q21 [11], 2q12.3 → q13 [12], 3q25.3 → q29 [13], 7q11.23 [14], 13q14 [15], 15q11 → q13 [16], 17p11.2 → p12 [17] and 22q11.2 [18]. A case of “pure” 1p36 tetrasomy has not yet been reported, although complex rearrangements resulting in deletions, duplications and/or triplications for portions of 1p36 have been reported elsewhere, along with postulated mechanisms of formation [19,20]. Thus, our study provides a new resource for studying the mechanisms of microtriplication formation.

In general, a tetrasomy causes a similar but more severe phenotype than a trisomy of the same region. For example, 7q11.23 duplication causes Williams-Beuren syndrome (WBS). The first patient reported to have a 7q11.23 triplication had a similar but more severe WBS phenotype, with severe developmental delay, severe retardation in language and speech, behavioral problems, autistic features and mild dysmorphic features [14]. Girirajan et al. described a patient with a large 17p11.2p12 duplication, partial 17p11.2p12 triplication and 17q11.2q12 deletion [17]. The severe phenotypic features in the patient included heart defects, seizure, thoracolumbar fistula and malignant hyperthermia. They attributed the disease-causing events to an increased copy number of dosage-sensitive genes in 17p11.2p12. We found that the clinical phenotypes of our patient with microtriplication 1p36 were similar to our other two patients with monosomy 1p36 (Table 1). These phenotypes included intellectual disability, developmental delay, feeding difficulties, hyperactivity and seizures. The two reported cases of 1p36 trisomy [5] had similar phenotypes to those in our patients with 1p36 tetrasomy and 1p36 deletion, but the clinical symptoms (psychomotor delay, intellectual disability, speech defects) were relatively mild.

The reason why 1p36 triplication causes more severe phenotype than 1p36 duplication, the dosage effect of gene expression in this region would be one of the possibilities. For example, craniosynostosis has been reported in patients with deletions and duplications/triplications of a gene on 1p36 that plays a role in regulating cranial suture closure [21]. In this 1p36 microtriplication patient, we found a *de novo* 5.28 Mb microtriplication at 1p36.33p36.32, involving 152 genes and 49 OMIM morbid genes. We hypothesize that the dosage effect of gene is related with the clinical phenotype of the patient. We selected four OMIM genes *B3GALT6*, *MIB2*, *PEX10*, *PANK4* which were expressed in the blood to test the hypothesis, and the results showed the patient had 3 to 6 times as higher expression levels than that in the control, which provided an evidence that overexpression of the specific genes might be related the specific phenotype of 1p36 microtriplication.

## Conclusions

Our study shows the first case of 1p36 tetrasomy due to a pure microtriplication in a patient with severe intellectual disability and seizures. The study provides a new resource for studying the mechanisms of microtriplication formation, and provides an evidence that overexpression of the specific genes might be related the specific phenotype of 1p36 microtriplication.

## Consent

Written informed consent was obtained from the parents of the patients for publication of this Case report and any accompanying images. A copy of the written consent is available for review by the Editors-in-Chief of this journal.

## Additional file

Additional file 1:
**Two 1p36 microdeletion syndrome patients in the study.**
