# Supplementary material for: The first patient with a pure 1p36 microtriplication associated with severe clinical phenotypes
Source: Mol Cytogenet. 2014 Oct 3;7:64. doi: 10.1186/s13039-014-0064-9 (PMC4198684; doi:10.1186/s13039-014-0064-9)

**Two 1p36 microdeletion syndrome patients in the study**

**Case 1 (Fig.A,B)**

The patient was an 8-year-old girl. She had been delivered by caesarian section after a full-term pregnancy. She was the first and only child of a 28-year-old father and a 26-year-old mother, both of whom were healthy and non-consanguineous. She had been hospitalized several times because of colds and fevers. She could say only two words, dad and mom, at 2 years of age. She had been hospitalized because of a seizure at the age of 7 years. She was obese (weight, 32 kg; height, 118 cm; BMI, 23 kg/m^2^ (>97th centiles)) [[Ma](http://www.ncbi.nlm.nih.gov/pubmed?term=Ma J%5BAuthor%5D&cauthor=true&cauthor_uid=20359376) et al., 2010]. She had microcephaly (head circumference, 50 cm). She had a dysmorphic face, with strabismus, hypertelorism, low hairline, ear malformations, broad nasal bridge, small mouth, thin upper lip and pointed chin (Fig. 1C, 1D; Table 1). She had a webbed neck, short limbs, wrinkled hands and feet, clinodactyly and cubitus valgus, but normal palm prints. The main clinical manifestation was mental retardation. She could only understand some simple sentences and could not express herself. She had behavioral abnormalities and was not very friendly.

**Case 2 (Fig.C,D)**

Another 8-year-old girl was brought to our clinic. She had been born at 38 weeks of gestation to healthy, unrelated parents. She was the first child of a 28-year-old father and a 27-year-old mother. She had a jaundice at birth. She was obese (weight, 24.45 kg; height, 109.9 cm; BMI, 20.24 kg/m^2^ (>97th centiles)) [[Ma](http://www.ncbi.nlm.nih.gov/pubmed?term=Ma J%5BAuthor%5D&cauthor=true&cauthor_uid=20359376) et al., 2010] and had microcephaly (head circumference, 49.5 cm). She had an expressionless face with hypertelorism, low hairline, ear malformations, broad nasal bridge small mouth, and pointed chin (Fig. 1E,1F; Table 1). She had a short neck, pectus carinatum, short limbs, wrinkled hands and feet, clinodactyly and scoliosis. The main clinical features were mental retardation, speech delay, hypotonia, aggressive behavior and poor expressive ability. Magnetic resonance imaging of the brain showed hypoplasia of the white matter. Ultrasonography of the uterus, liver, gallbladder, spleen, pancreas, kidneys and heart showed no abnormalities.


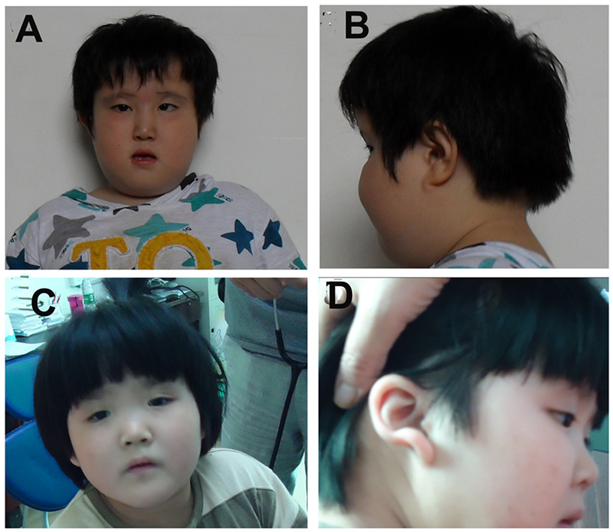

Supplement: Additional file 1: — Two 1p36 microdeletion syndrome patients in the study. [file 13039_2014_64_MOESM1_ESM.doc]
